# Case of Constipation of the Bowels, Removed by External Friction

**Published:** 1806-04

**Authors:** 

**Affiliations:** Surgeon, Walsall


### CASE of CONSTIPATION of the BOWELS, removed by EXTERNAL FRICTION;

*By* Mr. WEAVER, *Surgeon, Walsall.*

THOUGH the patient fell a victim to the complaint, yet it does not tend to disparage the means used. The child had been afflicted many days, previous to every recourse to medical aid; no evacuation had taken place for the space of ten days, at the expiration of which I was applied to, and found the child verging upon the precipice of eternity. The abdomen was extremely tense, upon which a blister was applied, having first premised the warm bath; she had frequent inclination to stool, accompanied with tenesmus, nausea, and vomiting: the rectum and colon were completely blocked up with indurated fæces, which her father occasionally removed with an instrument of his own manufacturing. Enemas of various kinds were used, but ineffectual; also castor oil, in conjunction with the usual remedies on similar occasions, as calomel, with and without opium, cathartic ext. &c. all to no effect. Fourteen days had now elapsed without being capable of affording the least assistance. When the regular mode of practice is rendered abortive by the obstinacy of the disease, we are frequently obliged to have recourse to the most plausible theory, though it may not have been sanctioned by the success of practical experience. I recollect the late Dr. Johnston, of Worcester, ordering immense quantities of hydrargy. pur. as a dernier resort, in similar cases; but the dictates of reason must ever revolt at a theory, so replete with mechanical ideas. That the specific gravity of quicksilver might be sufficiently ponderous, to force its way through a perpendicular tube, where no adequate resistance could be made, cannot admit of a doubt; but in the intestinal canal, which abounds with numerous circumvolutions, the *vis inertia* would unavoidably resist the impulse of the quicksilver.

After the present digression, permit me to observe that I recollected reading of a case of Constipation that was entirely removed (and the patient survived) by stimulating the intestines to action, through the medium of the absorbent system, but did not recollect the medicines used; however, the following were tried:

℞. Ung.

℞. Ung. ceræ. flav. ℥ß. Calomel, ppt. ʒij. Antim. tart. ℈i. Pulv. opii, gr. x. ft. liniment.

A fourth part of which was well rubbed upon the abdomen (where the blister had been applied) 4tis horis. After the friction had been used twice, a large quantity of fæces, intermixed with purulent matter was voided. The child died the morning following.

Quere. Whether the absorbents could have been acted upon in so short a space of time, so as to have procured the evacuation ? or if the near approach of death might not have had a tendency to cause that relaxation of the system which ensued prior to her dissolution ?

Quere. If the plan had been adopted in the early stage of the complaint, might it not have been productive of beneficial effects ?

